# AMPK Activation Serves as a Common Pro-Survival Pathway in Esophageal Adenocarcinoma Cells

**DOI:** 10.3390/biom14091115

**Published:** 2024-09-04

**Authors:** Niamh McNamee, Pavithra Rajagopalan, Aya Tal-Mason, Samuel Roytburd, Uma M. Sachdeva

**Affiliations:** Division of Thoracic Surgery, Massachusetts General Hospital, Boston, MA 02114, USA

**Keywords:** esophageal adenocarcinoma, AMPK, metabolism, chemoresistance, patient-derived organoids

## Abstract

Esophageal adenocarcinoma (EAC) is a subtype of esophageal cancer that is difficult to treat, with overall poor survival and frequent recurrence despite curative-intent treatment strategies. There is limited understanding of EAC resistance mechanisms to chemotherapy or radiation. We have found that the AMP-activated protein kinase (AMPK) can serve a pro-survival function in EAC cells in response to cytotoxic treatments. Treatment with the IL-6 inhibitor tocilizumab, which previously has been shown to inhibit EAC organoid growth, resulted in the activation of AMPK in the OE33 EAC cell line, which was accompanied by a decrease in MTORC1 signaling and an increase in oxidative mitochondrial metabolism, both known downstream effects of AMPK activation to promote cell survival under conditions of metabolic stress. This increase in oxidative metabolism was abrogated in cells with a genetic knockdown of AMPK expression. Furthermore, we found that AMPK was activated in OE33 cells following treatment with cisplatin or ionizing radiation. Treatment with the AMPK inhibitor Compound C or genetic knockdown of AMPK expression enhanced cell death in a synergistic manner with chemotherapeutics or ionizing radiation. These findings were recapitulated in human patient-derived EAC organoids, suggesting that AMPK may be a common pro-survival mechanism to confer treatment resistance in EAC and may serve as a novel target to enhance the efficacy of current and future treatment strategies.

## 1. Introduction

The metabolic activity of cancer cells can be exploited as a potential anti-cancer target. By preventing cancer cells from making the necessary biomolecules and metabolically adapting to their environment, cell survival may be disrupted with metabolic inhibitors. AMP-activated protein kinase (AMPK) is one of the main regulators involved in maintaining energy balance in the cell, and acts as a bioenergetic switch. When intracellular energy is low, as detected by a high ADP/ATP ratio, the AMPK catalytic subunit becomes activated via phosphorylation, leading to the upregulation of catabolic pathways to enhance intracellular ATP levels, including respiration and β-oxidation, while downregulating anabolic pathways such as protein and lipid synthesis [1]. AMPK can have dual roles in cancer cells, with some studies demonstrating that AMPK can serve a tumor-suppressive function to prevent cancer growth, such as in prostate cancer [2]. In other metabolic contexts, AMPK can promote the progression of tumors through the promotion of cell survival, and its inhibition may be a viable target in these contexts [3,4]. Cancer cells have been shown to exploit AMPK signaling as a pro-survival mechanism. AMPK becomes activated under nutrient-deprived conditions, such as limited glucose supply or hypoxia [5], promoting cancer cell survival through metabolic adaptation and mitochondrial biogenesis [6]. This metabolic plasticity has also been shown to drive chemoresistance in various cancer types [7].

Esophageal cancer comprises two main histologic subtypes, adenocarcinoma (EAC) and squamous cell carcinoma (ESCC). EAC differs from ESCC in its geographical prevalence, glandular phenotype, molecular signature, and its pathogenic risk factors such as obesity and Barrett’s Esophagus [8,9]. Esophageal cancer survival remains poor, with a 5-year survival rate of only 20% [10]. Although EAC and ESCC seem to represent different disease processes, clinical treatment regimens have historically been the same, including chemoradiotherapy followed by resection for locally advanced disease [11], though consistently improved responses have been seen for ESCC relative to EAC with this approach. EAC tumors have also been shown to have increased heterogeneity [12], which likely contributes to their more resistant phenotype [13] and underscores the need for improved therapeutics specifically targeting EAC tumors based on their unique cell physiology.

In this study, we aimed to investigate whether AMPK activation may serve as a mechanism by which EAC cells adapt to chemotherapy and radiation treatments to allow for metabolic adaptation, and whether AMPK inhibition may represent a potential new treatment strategy to enhance the efficacy of chemotherapy and radiation for EAC. Tocilizumab is an IL-6R inhibitor that has been shown to inhibit EAC cell growth [14]. We therefore investigated the effects of tocilizumab treatment on cell metabolism and AMPK pathway activation in OE33 EAC cells. We found that treatment with tocilizumab was associated with AMPK activation, and treatment with tocilizumab increased EAC oxidative metabolism in an AMPK-dependent manner. Furthermore, we found that AMPK was activated by chemotherapeutic cisplatin and by ionizing radiation, suggesting that AMPK may be commonly upregulated by EAC cells in response to stress. By inhibiting AMPK both chemically and through genetic knockdown, we were able to prevent AMPK-driven metabolic changes and synergistically increase cell death in EAC cell lines and patient-derived organoids (PDOs) treated concurrently with chemotherapy agents and ionizing radiation. These results suggest that concurrent AMPK inhibition may improve the efficacy of current treatment strategies.

## 2. Materials and Methods

### 2.1. Cell Culture

EAC cell lines OE19 and OE33 were obtained from Dr. Anil Rustgi under Material Transfer Agreement (MTA) 2021A001511 and grown in RPMI-1640 medium (Corning Inc., Corning, NY, USA; Cat. #: 10-040-CV) supplemented with 10% Heat-Inactivated Fetal Bovine Serum (R&D systems, Minneapolis, MN, USA; Cat. #: S11550H) and 1% Pen-Strep-Glutamine (Gibco, Thermo Fisher Scientific, New York, NY, USA; Cat. #: 10378-016). Details regarding these established EAC cell lines are provided in Supplemental Table S1.

### 2.2. Chemicals and Reagents

Drugs used in this study include tocilizumab (Selleck Chemicals, Houston, TX, USA; Cat. #: A2012), cisplatin (Sigma-Aldrich, St. Louis, MO, USA; Cat. #: P4394), paclitaxel, and Compound C (Sigma-Aldrich, St. Louis, MO, USA: Cat. #: 171260) at the concentrations indicated. IgG1κ (Millipore sigma, Burlington, MS, USA: Cat. #: I5154) was used as control for tocilizumab. Antibodies for p-AMPK (#2535), AMPK (#25320), p-ACC (#3661), ACC (#3662), p-S6 (#4858), and S6 (#2217) were purchased from Cell Signaling Technologies (Danvers, MA, USA). β-actin (Sigma-Aldrich, St. Louis, MO, USA: Cat. #: A5316) was used as loading control.

### 2.3. Western Blot

Cellular protein was prepared using RIPA buffer (Sigma-Aldrich, St. Louis, MO, USA: Cat. #: R0278) with protease inhibitors (Roche, Basel, Switzerland; Cat. #: 11697498001) and phosphatase inhibitors (Roche, Basel, Switzerland: Cat. #: 4906845001). The protein was resolved on 4–15% min-Protean TGX Pre-cast gels (Bio-Rad Laboratories, Waltham, MA, USA; Cat. #: 4561083) and transferred onto PVDF membranes (Bio-Rad Laboratories, Waltham, MA, USA: Cat. #: 1704157). The membranes were blocked using EveryBlot blocking buffer (Bio-Rad Laboratories, Waltham, MA, USA: Cat. #: 12010020) for 1 h followed by incubation with primary antibodies overnight. Secondary antibodies, anti-rabbit IgG (Li-Cor Biosciences, Lincoln, NE, USA; Cat. #: 926-32213) and anti-mouse IgG (Li-Cor Biosciences, Lincoln, NE, USA: Cat. #: 926-68070), were incubated at room temperature for 1 h before imaging using the ChemiDoc system (Bio-Rad Laboratories). Original western blots can be found in Supplementary Materials.

### 2.4. Seahorse Flux Analysis

Cells were seeded in 96-well Seahorse flux bioanalyzer plates and allowed to attach overnight. For treatment with tocilizumab or IgG control, cells were treated for 24 h prior to analysis. On the day of analysis, the medium was changed to XF RPMI medium (pH 7.4) (Agilent Technologies, Lexington, MA, USA; Cat. #: 103576) containing 10 mM glucose (Agilent Technologies, Lexington, MA, USA: Cat. #: 103577), 2 mM glutamine (Agilent Technologies, Lexington, MA, USA: Cat. #: 103579), and 1 mM pyruvate (Agilent Technologies, Lexington, MA, USA: Cat. #: 103578), and the cells were placed in a non-CO_2_ incubator for 1 h. A Mito Stress Test was performed with 2.5 μM oligomycin (Sigma-Aldrich, St. Louis, MO, USA: Cat. #: 75351), 1 μM FCCP (Sigma-Aldrich, St. Louis, MO, USA: Cat. #: C2920), 1 μM rotenone (Sigma-Aldrich, St. Louis, MO, USA: Cat. #: R8875), and 1 μM antimycin A (Sigma-Aldrich, St. Louis, MO, USA: Cat. #: A8674). Data were normalized with protein concentration or cell number. OCR and ECAR rates were analyzed using the Seahorse Wave Desktop software 2.6 (Agilent Technologies).

### 2.5. siRNA Transfection

OE19 and OE33 were seeded in 6-well plates and allowed to attach overnight. ON-TARGET plus PRKAA1 (Dharmacon, Lafayette, CO, USA; Cat. #: L-005027-00-0005) and PRKAA2 (Dharmacon, Lafayette, CO, USA: Cat. #: L-005027-00-0005) SMARTpool siRNA were diluted in OptiMEM (Life Technologies, Carlsbad, CA, USA Cat. #: 31985-070) and mixed with RNAiMax lipofectamine (Life Technologies; Carlsbad, CA, USA: Cat. #: 13778075). ON-TARGET Plus non-targeting pool (Dharmacon, Lafayette, CO, USA: Cat. #: D-001810-10-05) was used as knockdown control. Cells were incubated with siRNA-lipofectamine mix for 6 h before replacing the medium. Cells were seeded for further experiments on the following day. AMPK knockdown was determined by polymerase chain reaction (PCR).

### 2.6. Quantitative Real Time PCR

RNA was extracted from cells using the RNeasy Plus Mini Kit (Qiagen, Hilden, Germany, Cat. #: 74134) and 1 µg of RNA was converted to cDNA using a High-Capacity RNA-to-cDNA kit (Applied Biosystems, Beverly Hills, CA, USA; Cat. #: 4387406). RNA was diluted 2.5 times with nuclease-free water (IDT, Newark, NJ, USA; Cat. #: 11-05-01-14) and PCR was performed using PowerUp SYBR Green (Applied Biosystems, Beverly Hills, CA, USA: Cat. #: A25741) as per the manufacturer’s instructions on QuantStudio 3 Real-Time PCR. AMPK primer sequences: *PRKAA1*; F:TGCGTGTACGAAGGAAGAATCC R:TGTGACTTCCAGGTCTTGGAGTT.

### 2.7. Patient-Derived Organoids

The PDOs used in this study were previously generated and published by the Rustgi lab [15,16], and received as a generous gift to our lab under Material Transfer Agreement 2021A001511. The PDOs were generated from endoscopic tissue biopsies from consenting patients by single-cell dissociation and seeding in Matrigel (MG), as published by the Rustgi lab [15,16]. Patient characteristics for each PDO are provided in Supplemental Table S2, as previously published [16].

For our experiments, the PDOs were placed in MG (Corning Inc., Corning, NY, USA: Cat. #: 254234) and grown in Advanced DMEM as previously described [15]. To passage, MG was dislodged, and organoids were placed in dispase (Corning Inc., Corning, NY, USA; Cat. #: 354235) at 37 °C for 10 min to remove MG. Organoids were spun down and resuspended in 0.25% trypsin (Gibco, Thermo Fisher Scientific, New York, NY, USA: Cat. #: 25200056) for 10 min. Soybean trypsin inhibitor (Sigma-Aldrich, St. Louis, MO, USA: Cat. #: T91280) (STI) was used to stop trypsin reaction and cells were centrifuged and resuspended in basal medium (Advanced DMEM) with GlutaMAX (Gibco, Thermo Fisher Scientific, New York, NY, USA: Cat. #: 35050061), HEPES (Gibco, Thermo Fisher Scientific, New York, NY, USA: Cat. #: 15630106), and Anti-Anti (Gibco, Thermo Fisher Scientific, New York, NY, USA: Cat. #: 15-240-062)). Cells were counted and desired cell numbers seeded in 50 μL of MG in 24-well plates or 3 µL in 96-well plates.

### 2.8. Cytotoxicity Assay

CellTiter 96 Aqueous One Solution Cell Proliferation Assay (VWR International, Radnor, PA, USA; Cat. #: G3580) was used to determine cell viability as per the manufacturers’ instructions. Briefly, cells were seeded in 96-well plates and allowed to attach overnight. Following 72 h of drug treatment, CellTiter solution was diluted 1:5 in medium and 100 µL was added to each well. The cells were incubated for 1 h and absorbance was measured at 490 nm using a Tecan plate reader. For 3D cytotoxicity, OE33 and OE19 cells or PDOs were seeded in 3 µL of MG and allowed to establish organoids for 3–4 days before treatment. For AMPK knockdown, OE33 and OE19 cells were transfected with siRNA before plating cells in Matrigel for 3D culture. CellTiter-Glo 3D cell viability assay (Promega, Madison, WI, USA; Cat. #: G9681) was used as per the manufacturers’ instructions after 72 h.

### 2.9. Statistical Analysis

All results shown are from a minimum of three independent experiments. When two groups are compared, a Student’s *t*-test was used. For more than two groups, one-way ANOVA was used for statistical analysis. Data are shown as mean ± SEM. * *p* < 0.05, ** *p* < 0.01, *** *p* < 0.001.

## 3. Results

### 3.1. Il-6 Inhibition with Tocilizumab Activates AMPK and Downstream Targets

**Figure 1:**
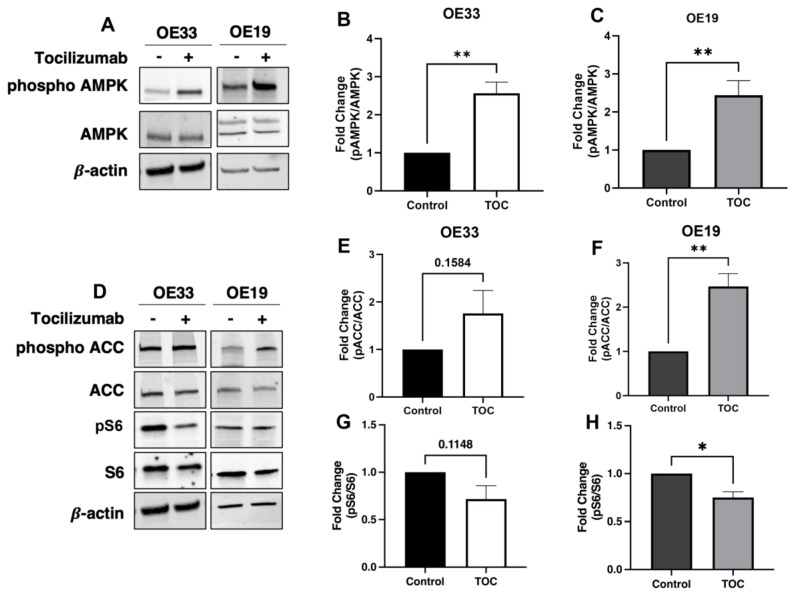
Tocilizumab induces AMPK activation in EAC cell lines. Western blot was used to determine levels of p-AMPK Thr172 (**A**) after treatment with tocilizumab in OE33 (100 µg/mL) and OE19 (500 µg/mL) cells, with quantification of the p-AMPK-to-total AMPK ratio by densitometry analyses (**B**,**C**). Downstream phosphorylation of ACC and S6 were analyzed by Western blot (**D**), and the phosphorylated fractions were calculated for both p-ACC (**E**,**F**) and p-S6 (**G**,**H**) by densitometry, with normalization to total ACC and S6 levels. β-actin was used as loading control for all samples. Graphs represent mean ± SEM of n = 3 experiments. * *p* > 0.05, ** *p* > 0.01.

The treatment of OE33 and OE19 cells with tocilizumab induced AMPK activation (Figure 1A), as demonstrated by an increase in AMPK phosphorylation at the activating threonine 172 site (Figure 1B,C) and the modulation of downstream effectors [17], including the increased phosphorylation of acetyl coA carboxylase 1 (ACC1) and the decreased phosphorylation of mammalian target of rapamycin complex 1 (MTORC1) targets (Figure 1D–H). 

### 3.2. Il-6 Inhibition with Tocilizumab Increases Oxidative Metabolism

**Figure 2:**
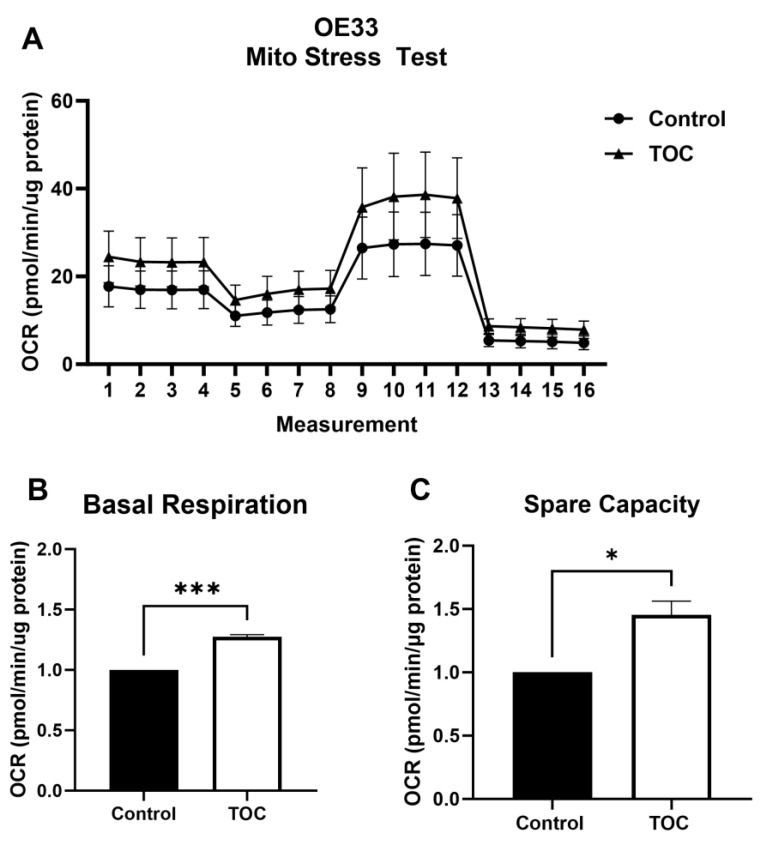
Tocilizumab induces oxidative metabolism in EAC cell lines. A Mito stress test was performed on OE33 cells treated with 100 µg/mL tocilizumab or IgG control (**A**). Basal respiration (**B**) and spare capacity (**C**) were quantified in tocilizumab and control-treated cells. Seahorse data were normalized to µg of protein. Graphs represent mean ± SEM of n = 3 experiments. * *p* > 0.05, *** *p* > 0.001.

In accordance with known AMPK-mediated effects on oxidative metabolism, tocilizumab concurrently increased the oxygen consumption rate in EAC cells (Figure 2A), with a significant increase in OE33 basal respiration (Figure 2B) and spare capacity (i.e., the difference between maximal respiration and basal respiration) (Figure 2C). Similar effects on OE19 cell metabolism were also observed (Supplemental Figure S1A–C). 

### 3.3. AMPK Knockdown Decreases Oxidative Metabolism in the Presence of Tocilizumab

**Figure 3:**
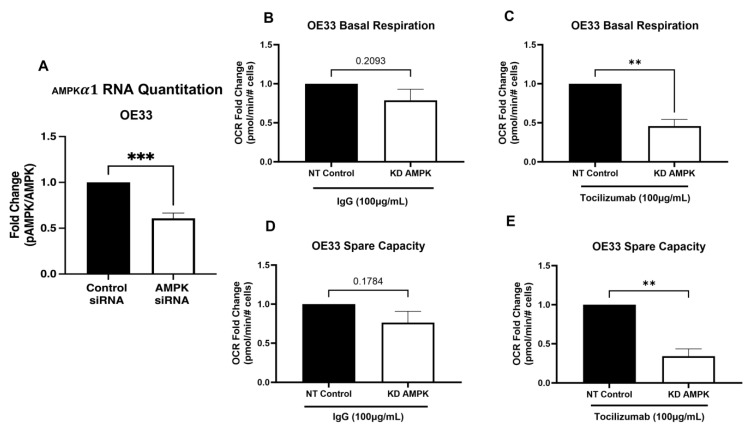
AMPK knockdown prevents tocilizumab-induced increase in oxidative respiration. AMPKα1 knockdown was confirmed following treatment with targeting or non-targeting siRNA by qPCR (**A**). OE33 cells were treated with 100 µg/mL IgG or tocilizumab for 24 h prior to Seahorse analysis measuring basal respiration (**B**,**C**) and spare capacity (**D**,**E**). Seahorse was normalized to cell number. Graphs represent mean ± SEM of n = 3 experiments. ** *p* > 0.01, *** *p* > 0.001.

To determine whether the increase in oxidative respiration and spare capacity following tocilizumab treatment was dependent on AMPK activation, we inhibited the expression of AMPK using siRNA knockdown in OE33 cells, achieving almost 50% reduction in AMPK transcript expression (Figure 3A). The knockdown of AMPK had no effect on basal respiration (Figure 3B) or spare capacity (Figure 3D) in control IgG-treated cells but resulted in the downregulation of oxidative metabolism in tocilizumab-treated cells (Figure 3C,E), indicating that enhanced oxidative respiration in the presence of tocilizumab is dependent on AMPK. Similar effects were seen in OE19 cells (Supplemental Figure S2A–E).

### 3.4. AMPK Signaling Is Activated upon Treatment with Chemotherapeutics and Radiation

**Figure 4:**
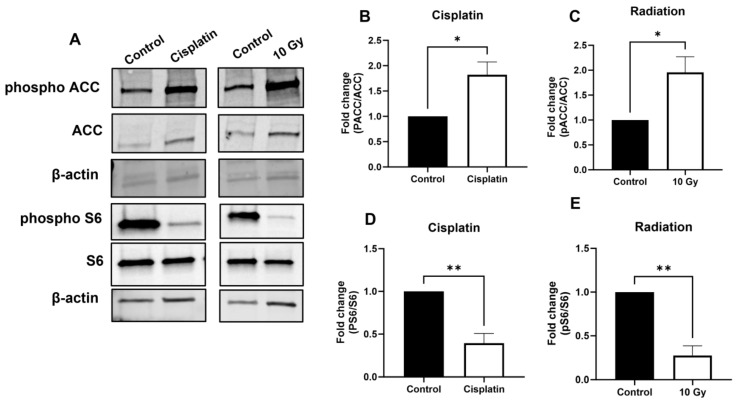
AMPK downstream signaling increased following treatment with cisplatin or radiation. OE33 cells were treated for 72 h with cisplatin (1 µM) or radiation (10 Gy). Representative blots demonstrating expression levels of P-ACC and P-S6 are shown (**A**). Densitometry quantification of P-ACC after cisplatin (**B**) or radiation (**C**), as normalized to total ACC levels. Densitometry quantification of P-S6 after cisplatin (**D**) or radiation (**E**), as normalized to S6 levels. β-actin was used as loading control. Graphs represent mean ± SEM of n = 3 experiments. * *p* > 0.05, ** *p* > 0.01.

We next sought to determine whether AMPK activation may represent a common pathway in response to treatment with chemotherapies or radiation in EAC cells. OE33 cells were treated with 1 µM cisplatin or 10 Gy ionizing radiation, which resulted in increased AMPK activation as demonstrated by the immunoblot of canonical downstream targets (Figure 4A). Cisplatin (Figure 4B) and radiation (Figure 4C) both induced an increase in ACC phosphorylation and a significant decrease in S6 phosphorylation (Figure 4D,E), indicative of AMPK activation.

### 3.5. AMPK Inhibition Promotes the Cytotoxic Effect of Chemotherapies and Radiation in a Synergistic Manner

**Figure 5:**
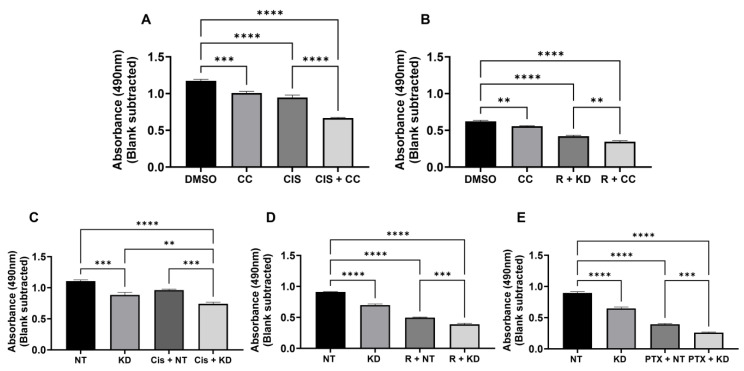
Inhibition of AMPK in combination promotes synergistic cytotoxic effects. OE33 cells were treated with cisplatin (Cis, 1 µM), radiation (10 Gy), or paclitaxel (Ptx, 1 nM) in combination with Compound C (CC, 1 µM) (**A**,**B**) or AMPK siRNA knockdown (**C**–**E**), and viability was assessed after 72 h. NT = NT siRNA; KD = KD siRNA; and R = 10 Gy. Graphs are representative of n = 3 experiments. ** *p* > 0.01; *** *p* > 0.001; **** *p* > 0.0001 by one-way ANOVA.

Given the role of AMPK activation in promoting cell survival in the setting of metabolic stress [6,18], we sought to determine whether AMPK inhibition may enhance cell death after treatment with chemotherapeutic agents and ionizing radiation. OE33 cells were treated with the AMPK inhibitor Compound C in combination with either cisplatin (Figure 5A) or radiation (Figure 5B) and demonstrated significant decrease in viability when compared to cells treated with cisplatin or radiation and saline control. Enhanced cell death was also observed in OE33 cells treated with cisplatin, paclitaxel, or radiation following the siRNA knockdown of AMPK expression (Figure 5C–E). Similar cytotoxic effects were seen in OE19 cells (Supplemental Figure S3A–E). Interestingly, a significant reduction in viability with AMPK knockdown or Compound C treatment alone was also observed, though these effects were enhanced in combination with chemotherapy or radiotherapy application.

### 3.6. Inhibition of AMPK Enhances Cytotoxic Effects of Chemotherapy and Radiation in Patient-Derived EAC Organoids

**Figure 6:**
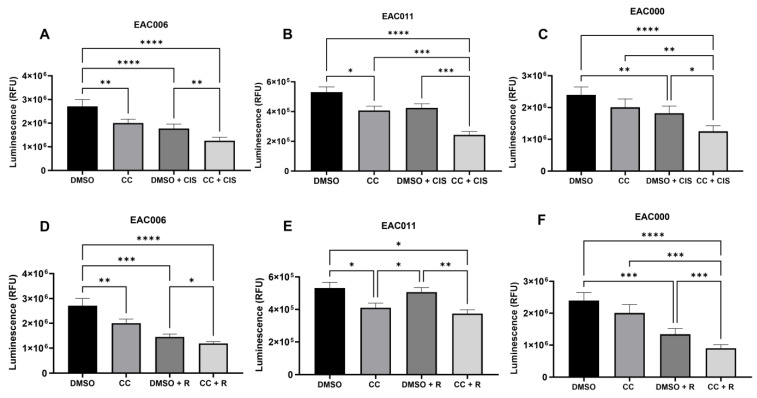
Inhibition of AMPK synergizes with cisplatin and radiation in 3D EAC PDOs. EAC PDOs were seeded and allowed to expand for 3–4 days prior to treatment with cisplatin (**A**–**C**) (50 µM for 006 and 000, 100 µM for 011) or radiation (**D**–**F**) (20 Gy) in combination with Compound C (10 µM) for 72 h. Graphs are representative of n = 5 (006), n = 4 (011), and n = 3 (000) experiments. R = 20 Gy. * *p* > 0.01; ** *p* > 0.01; *** *p* > 0.001; **** *p* > 0.0001. One-way ANOVA with matched analysis was performed.

To determine whether the promotion of cytotoxicity by AMPK inhibition could translate to real-world EAC patient tumors, three established and published patient-derived EAC organoid lines [16] were treated with cisplatin, paclitaxel, or radiation in combination with Compound C. All three PDO lines showed synergistic effects of Compound C with cisplatin (Figure 6A–C) or Compound C with ionizing radiation (Figure 6D–F). The combination of Compound C with paclitaxel also provided added benefit (Supplemental Figure S4A–C). Interestingly, two of the three PDO lines tested (PDOs 006 and 011) also showed a significant decrease in viability with Compound C alone (Figure 6A,D) though cytotoxicity was enhanced when Compound C was used in combination with standard treatments. 

## 4. Discussion

AMPK serves a pro-survival function in cells under conditions of stress [6]. Both cisplatin and ionizing radiation cause metabolic stress through the induction of DNA damage and the generation of reactive oxygen species [19,20]. Here, we have found that AMPK activation is a common mechanism by which EAC cells and PDOs respond to multiple cytotoxic agents that are either currently in use or currently being evaluated in preclinical settings. Furthermore, we have found that the inhibition of AMPK enhances the cytotoxic effects of standard-of-care treatment agents for EAC. These results suggest that AMPK activation may promote EAC resistance to treatments, and its inhibition may be a promising therapeutic approach to enhance the cytotoxicity of current and future treatments for EAC.

Depending on the context, AMPK has been shown to have both pro-survival and pro-apoptotic roles in cancer cells [21,22]. AMPK activation has been shown to enhance survival following nutrient deprivation in H1299 non-small-cell lung cancer cells [6]. AMPK activation has also been shown to be required for downstream oncogenic AKT signaling in breast cancer cells exposed to various stressors including hypoxia, glucose deprivation, and EGF treatment [21]. The knockdown of AMPK in breast cancer cells resulted in decreased migration and glucose metabolism [21].

Although no prior study has investigated the effects of AMPK manipulation on the growth or viability of EAC cells, the activation of AMPK has been found to have an inhibitory effect in ESCC cells, enhancing the sensitivity of several ESCC cell lines to cisplatin [22]. The natural product Silibinin, which activates AMPK, also demonstrated inhibitory effects on ESCC cells that were able to be reversed with AMPK inhibition [23]. In a separate study, AMPK inhibition by Compound C was shown to reduce radio-resistance in an ESCC cell line [24]. These studies suggest that the role of AMPK may differ in EAC versus ESCC, underscoring the need for more personalized and precise approaches to the treatment of these distinct esophageal cancer histologic subtypes.

Il-6 has been shown to promote EAC cell proliferation in vitro, promoting epithelial-mesenchymal transition (EMT) and the growth of EAC tumoroids in 3D culture, effects which were inhibited by treatment with tocilizumab [14]. Here, we treated OE33 and OE19 EAC cell lines with tocilizumab and observed an activation of AMPK signaling and its downstream effectors, including the inhibitory phosphorylation of ACC1 and a decrease in S6 ribosomal protein phosphorylation. Basal respiration, maximal respiration, and spare capacity, a measure of cell metabolic fitness, also increased after tocilizumab treatment, in accordance with AMPK’s known effects in promoting oxidative metabolism to improve cellular bioenergetics under conditions of metabolic stress [6].

To determine whether standard clinical agents may produce a similar effect in EAC cells, we demonstrated that downstream AMPK signaling also becomes activated when cells are treated with cisplatin or radiation, suggesting that EAC cells may activate AMPK as a common mechanism of survival. By inhibiting AMPK through genetic knockdown, we demonstrated that tocilizumab was no longer able to increase the oxidative metabolism of EAC cells, further implicating the role of AMPK activation in promoting cell survival under the stress of tocilizumab treatment. AMPK activation was also observed following treatment with cisplatin or ionizing radiation, and the inhibition of AMPK, either chemically or by genetic knockdown, enhanced the cytotoxicity of cisplatin and radiation. These results have translational applications, as we observed synergistic cytotoxic effects of Compound C when used in combination with cisplatin, radiation, and, to a lesser extent, paclitaxel in patient-derived EAC organoid lines.

The combination of AMPK inhibition with standard anti-cancer therapies has shown promise in prostate cancer, where cells treated with mitomycin C in combination with either genetic or chemical AMPK inhibition resulted in increased apoptosis in vitro [25]. Our data also indicate that AMPK inhibition alone reduces viability in EAC cells and PDOs, suggesting that EAC may be particularly dependent on AMPK activity to regulate its metabolic program. Further studies are required to determine whether this may reflect an underlying dependence on oxidative metabolism in EAC.

## 5. Conclusions

Taken together, our results indicate that AMPK inhibition may represent a viable adjunct treatment to improve cytotoxicity and overcome resistance that is frequently observed in EAC. We have demonstrated that AMPK is activated in response to various standard and preclinical treatments for EAC, resulting in enhanced oxidative metabolism, and that AMPK inhibition synergistically enhances the cytotoxic effects of chemotherapy and radiation treatments when used in combination.

## Data Availability

All data, cell lines, and reagents used in this study are available upon request or commercially available as indicated. There were no datasets generated in this study.

## Supplementary Materials

The following supporting information can be downloaded at: https://www.mdpi.com/article/10.3390/biom14091115/s1, Figure S1: Il-6 inhibition increases oxidative metabolism in OE19 cells; Figure S2: AMPK knockdown decreases oxidative metabolism of OE19 cells in the presence of tocilizumab; Figure S3: AMPK inhibition promotes the cytotoxic effect of chemotherapies and radiation in a synergistic manner in OE19 cells; Figure S4: Inhibition of AMPK provides added benefit with paclitaxel treatment in 3D EAC PDOs; Table S1: Characteristics of EAC cell lines; Table S2: Characteristics of EAC PDOs.
