# Supplementary material for: AMPK Activation Serves as a Common Pro-Survival Pathway in Esophageal Adenocarcinoma Cells
Source: Biomolecules. 2024 Sep 4;14(9):1115. doi: 10.3390/biom14091115 (PMC11429576; doi:10.3390/biom14091115)

Figure 1:
OE33 Full Gels

pAMPK

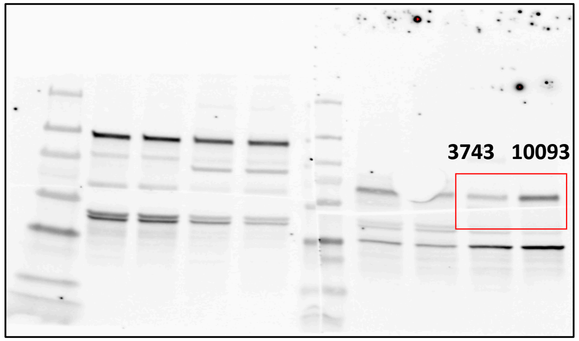

AMPK

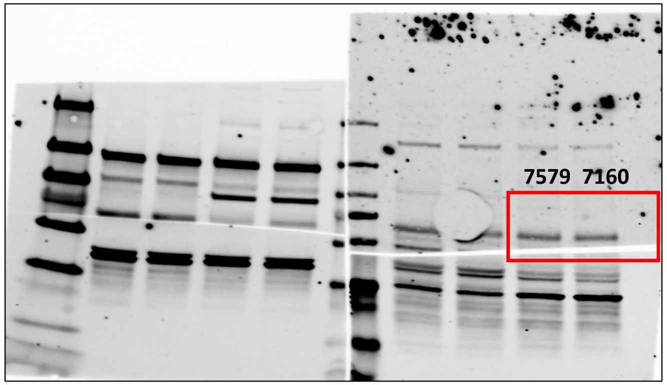

B-actin

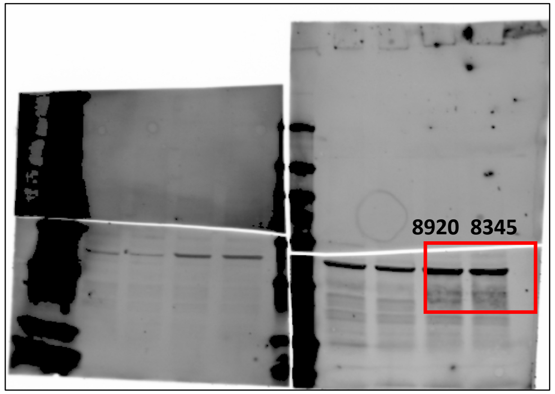

pACC

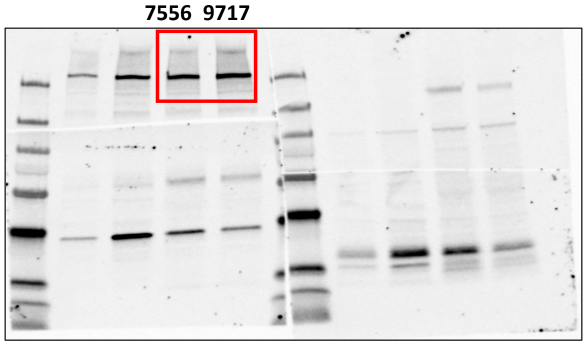

ACC

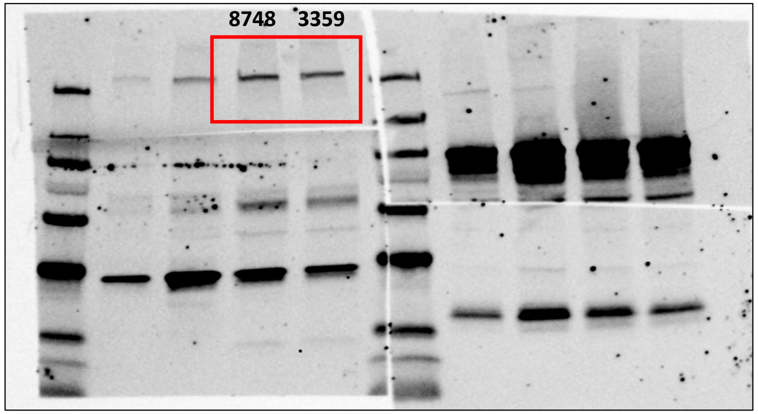

B-actin

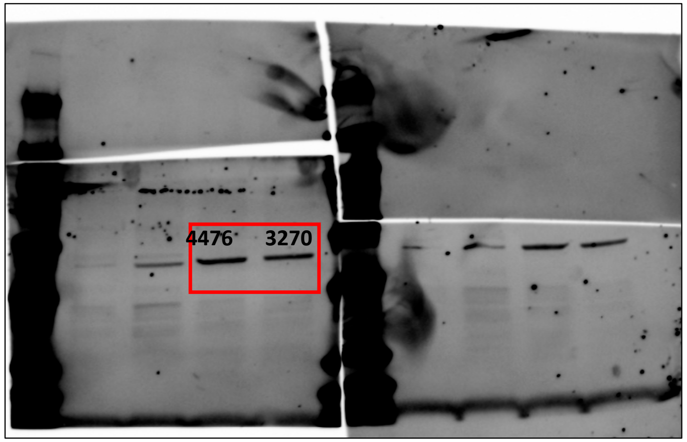

pS6

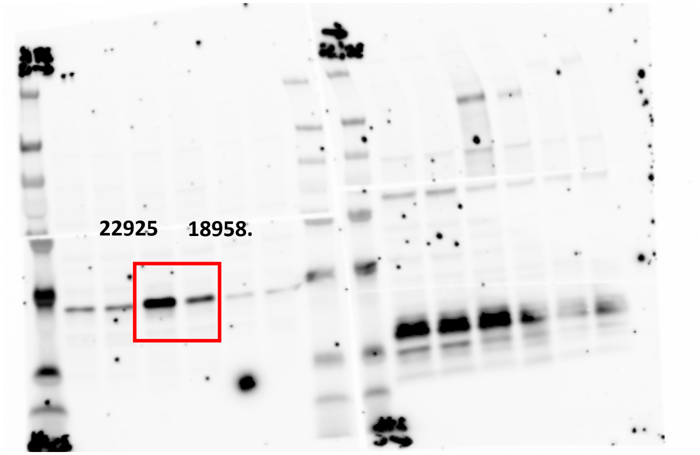

S6

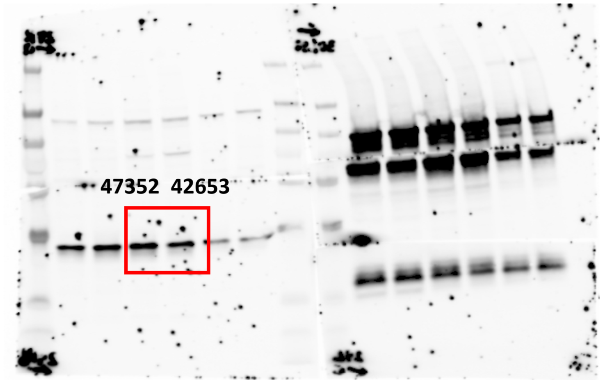

B-actin

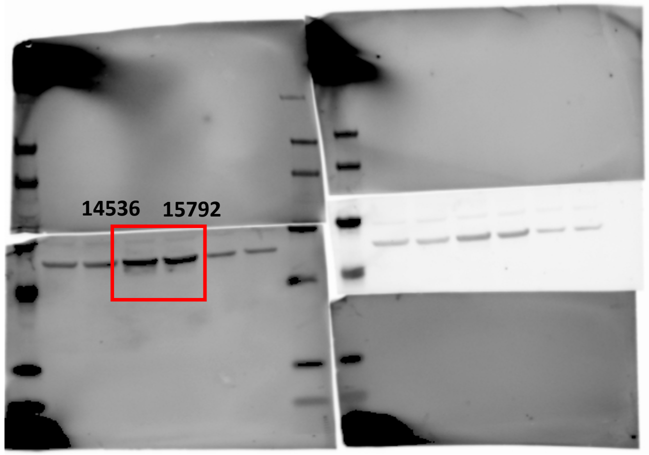

Figure 1:
OE19 Full Gels

pAMPK

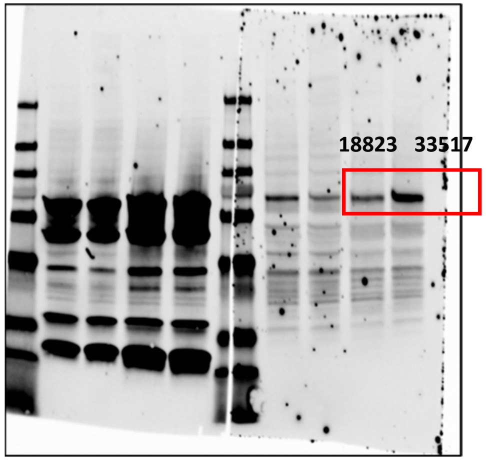

AMPK

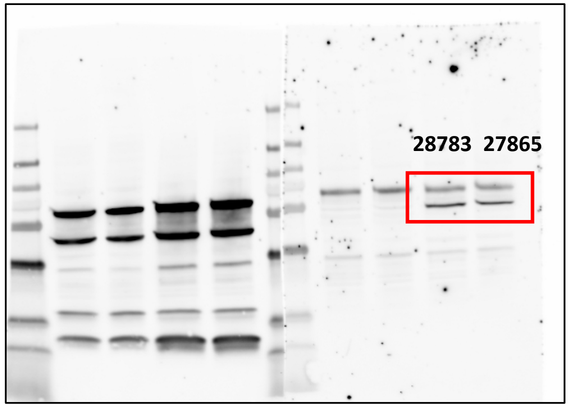

B-actin

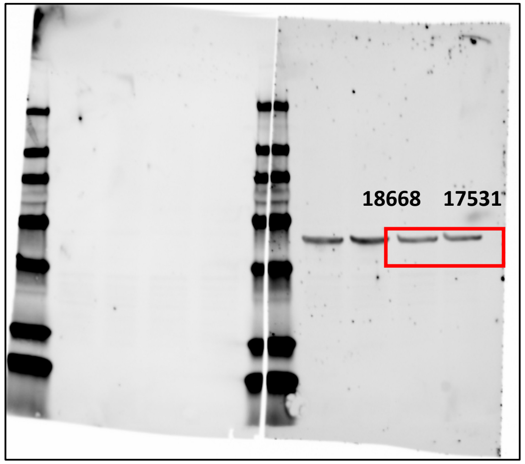

pACC

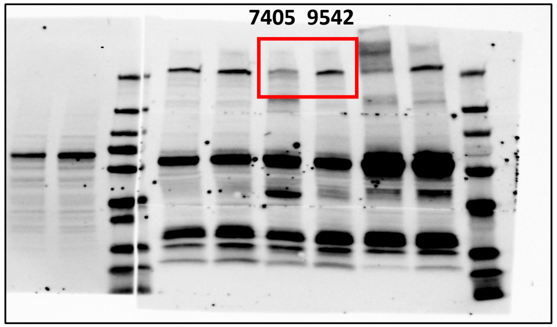

ACC

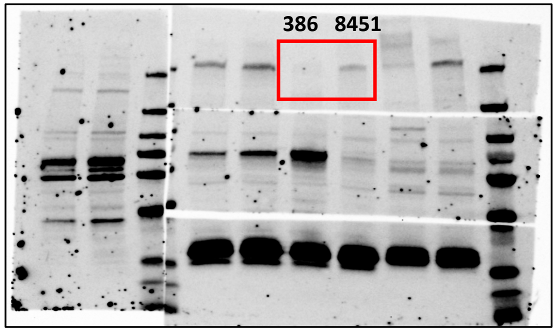

B-actin

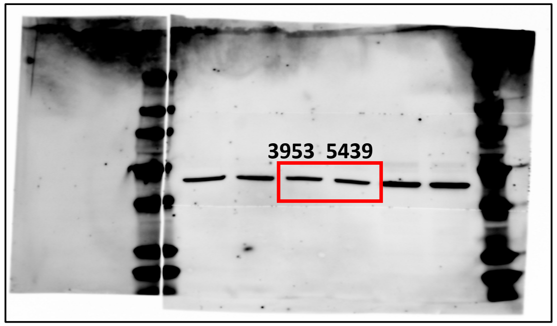

pS6

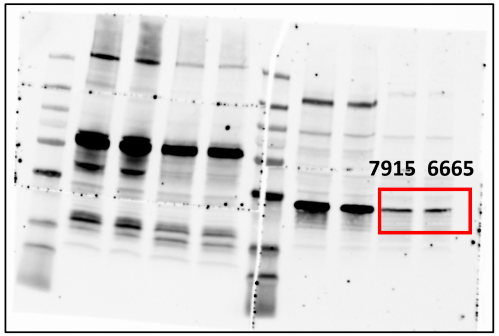

S6

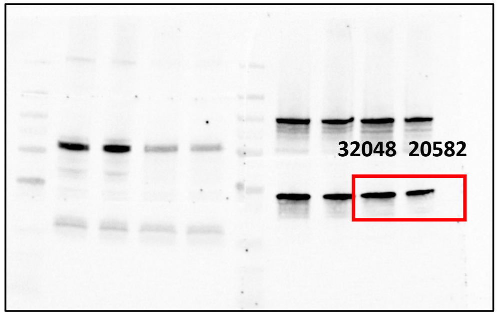

B-actin

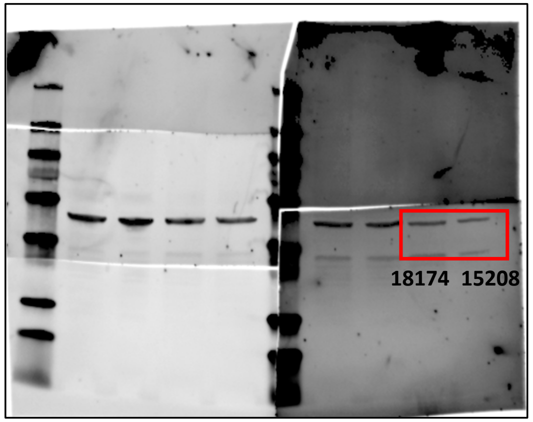

Figure 4:
OE33 Full Gels

ACC

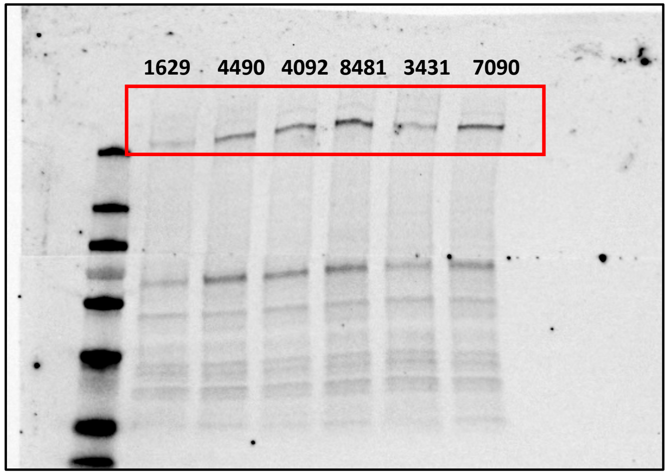

S6

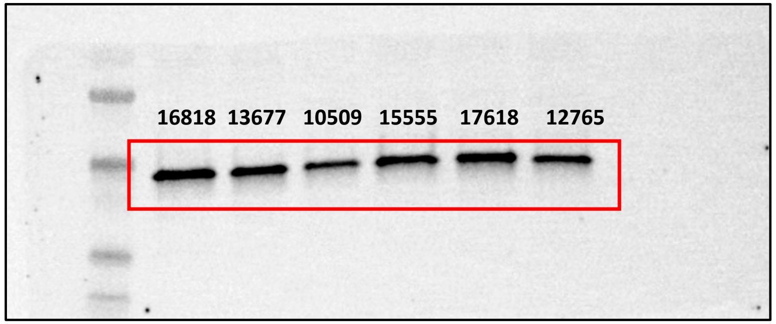

B-actin

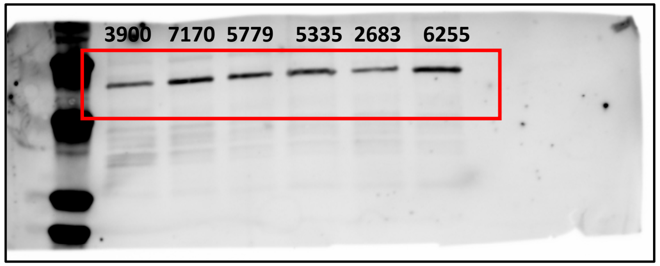

pACC

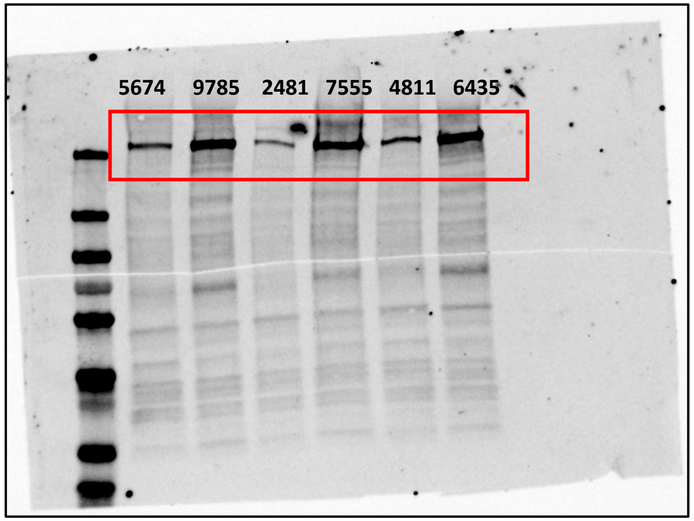

pS6

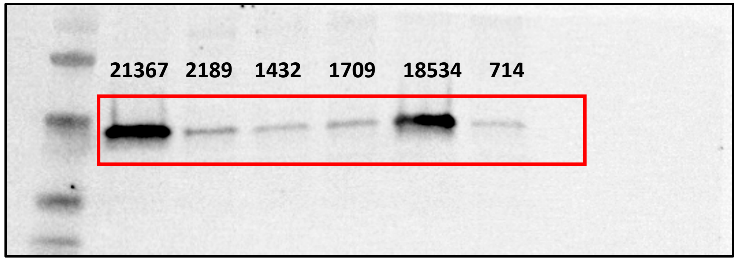

B-actin

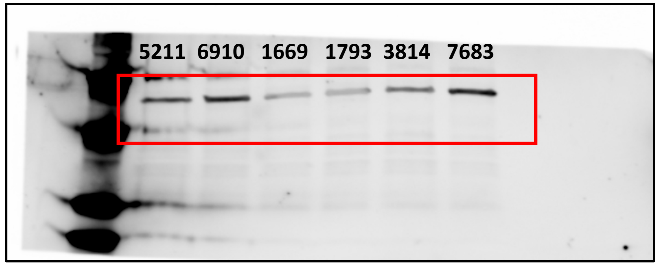

Supplement: Supplementary file 1 [file biomolecules-14-01115-s001.zip › AMPK Gel suppl..pdf]
