# Supplementary material for: AMPK Activation Serves as a Common Pro-Survival Pathway in Esophageal Adenocarcinoma Cells
Source: Biomolecules. 2024 Sep 4;14(9):1115. doi: 10.3390/biom14091115 (PMC11429576; doi:10.3390/biom14091115)
Supplement: Supplementary file 1 [file biomolecules-14-01115-s001.zip › biomolecules-3136962-supplementary tables.pdf]

Supplemental Table S1

	Location of tumor	Patient Gender	Patient Age	Pathological Stage (UICC)	Differentiation
**OE19 cell line**	Gastric Cardia	Male	72	III	Moderate
**OE33 cell line**	Lower Esophagus	Female	73	IIA	Poor

Table S1: Characteristics of EAC cell lines:
The following table lists characteristic features of cell lines used in this study that are available from the European Collection of Authenticated Cell Lines.

Supplemental Table S2

	**Patient Gender**	**Patient Age**	**Pathological Stage (pre-Tx)**	**Pathological Stage (post-Tx)**	**Differentiation**
**EAC006**	Male	79	T3N2M0	T2N2M0	Moderate
**EAC011**	Male	63	T3N1M0	T3N0M0	Poor
**EAC000**	Male	76	T2N0M0	T1bN0M0	Moderate

Table S2: Characteristics of PDOs:
The following table lists characteristic features of PDOs generated in the Rustgi lab and previously reported [16].
