# Supplementary figures and images for: AMPK Activation Serves as a Common Pro-Survival Pathway in Esophageal Adenocarcinoma Cells
Source: Biomolecules. 2024 Sep 4;14(9):1115. doi: 10.3390/biom14091115 (PMC11429576; doi:10.3390/biom14091115)

# SUPPLEMENTAL FIGURE S1

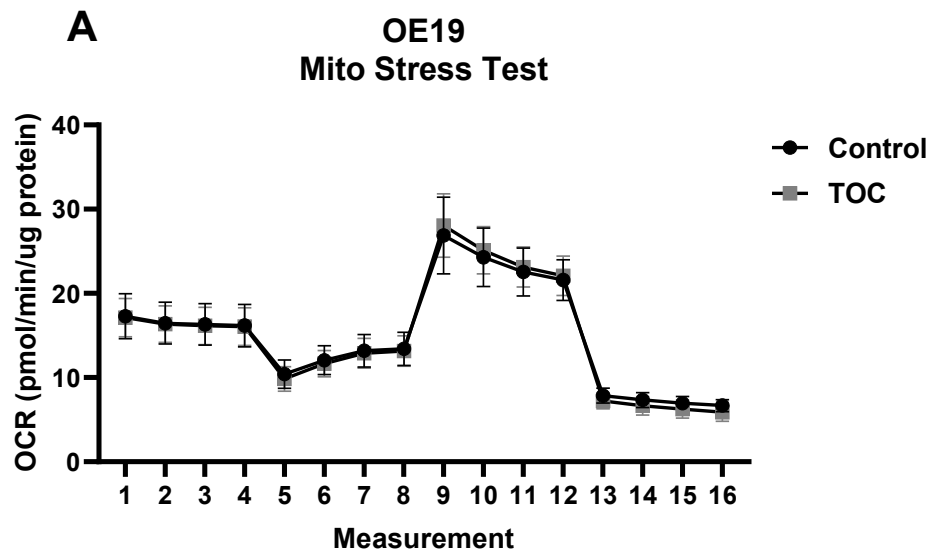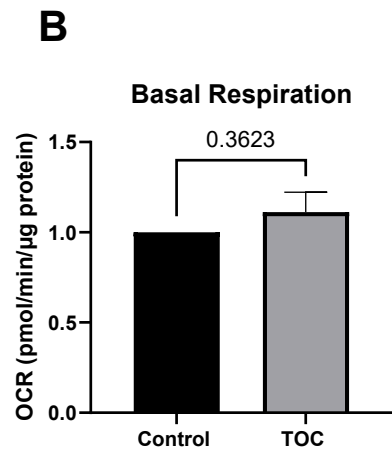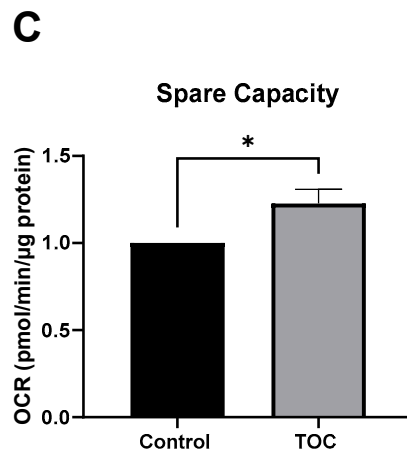

SUPPLEMENTAL FIGURE S2

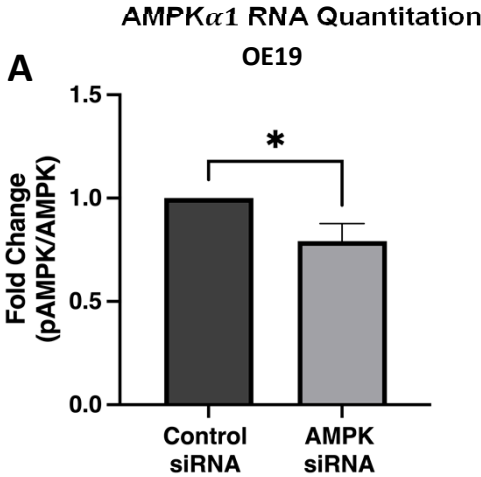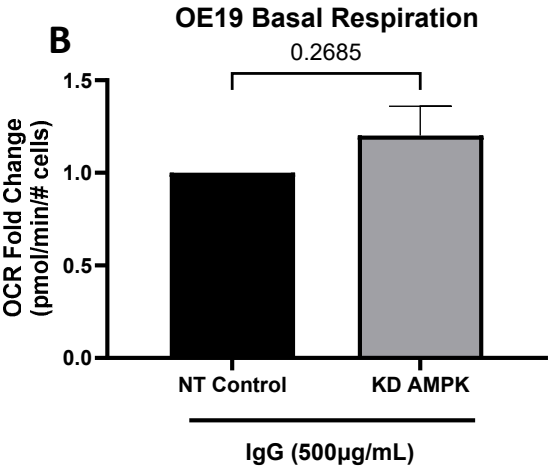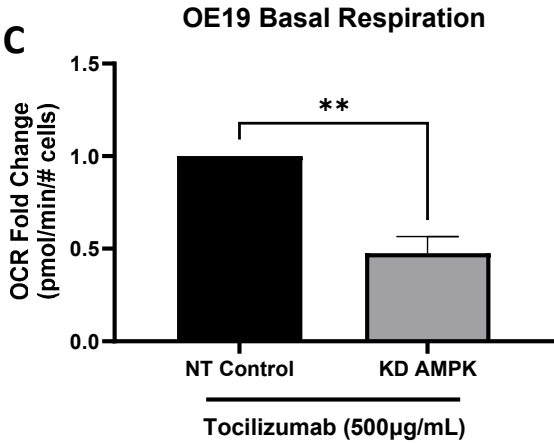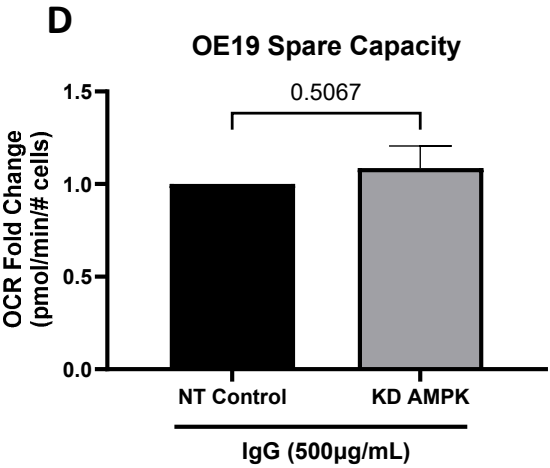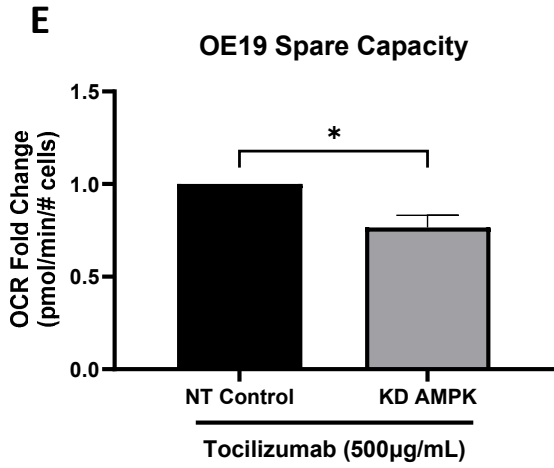

## Supplementary FIGURE S3

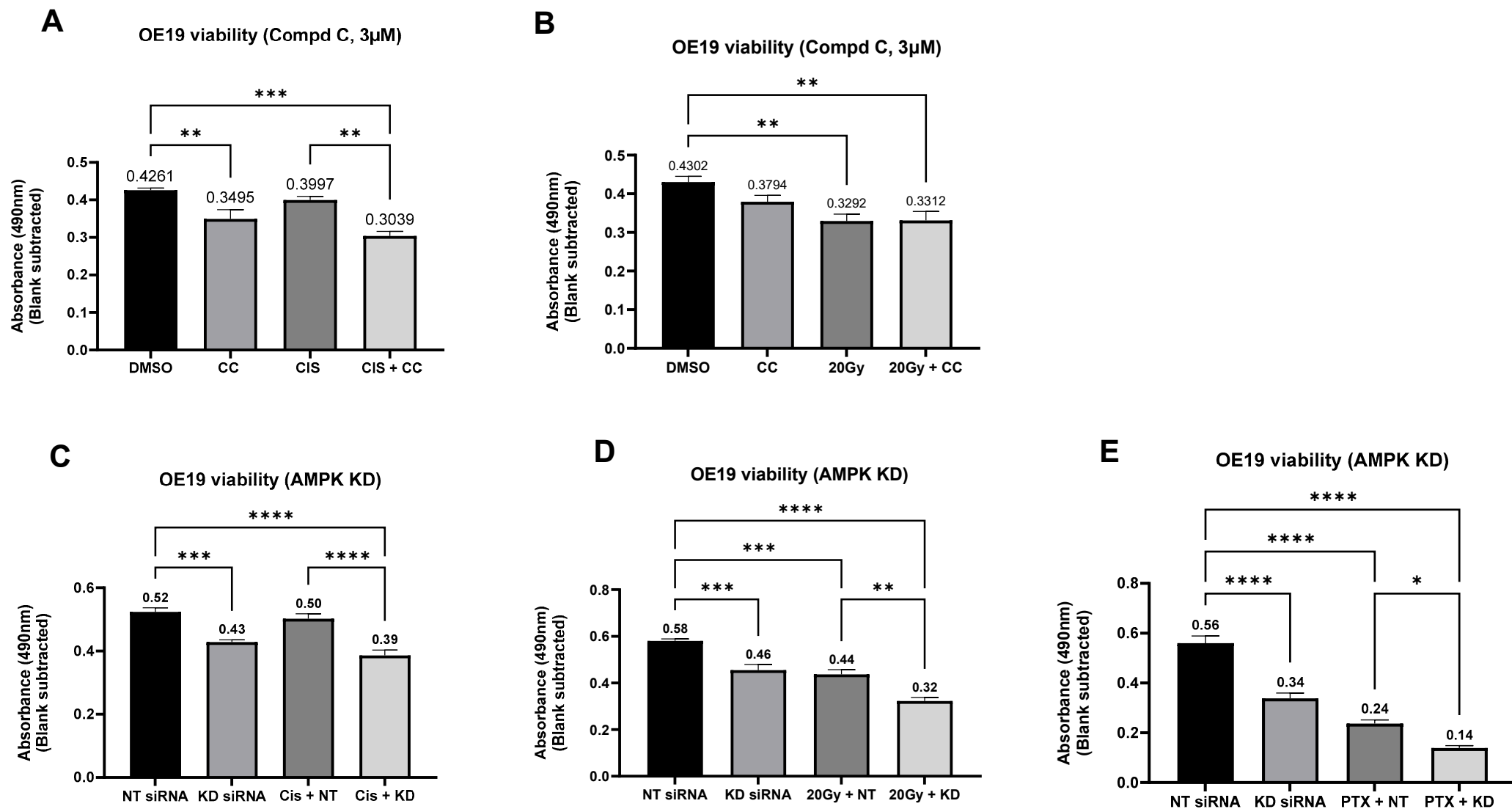

# Supplementary FIGURE S4

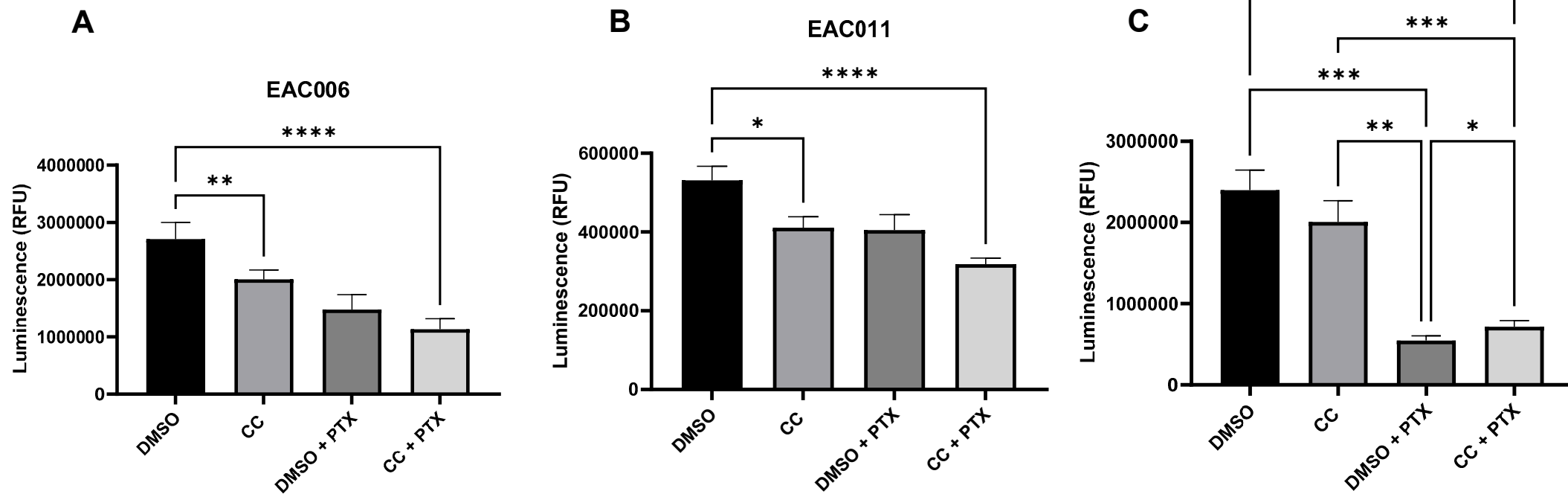

Supplement: Supplementary file 1 [file biomolecules-14-01115-s001.zip › AMPK_paper_Supple_figures.pdf]
